# An Unusual Presentation of Insert Dislocation and MCL Rupture in Unicompartmental Knee Replacement with 2 Years Postoperative Results: Does It Functional?

**DOI:** 10.1155/2019/2634738

**Published:** 2019-05-14

**Authors:** Ali Asma, Mehmet Erduran, Musa Eymir

**Affiliations:** ^1^Department of Orthopedics and Traumatology, School of Medicine, Dokuz Eylul University, TR-35340, Balçova, Izmir, Turkey; ^2^School of Physical Therapy and Rehabilitation, Dokuz Eylul University, TR-35340, Balçova, Izmir, Turkey

## Abstract

According to our knowledge, there is no prior article that reports functional results of medial collateral ligament (MCL) primary repair and insert change after MCL rupture and mobile-bearing dislocation as a late complication of unicompartmental knee replacement (UKR). Firstly, 63-year-old woman was treated with UKR due to anteromedial knee osteoarthritis of the right knee joint. 1 year after UKR surgery, she suffered from MCL rupture and mobile-bearing dislocation because of falls while getting on a public bus, and therefore, secondly, she was operated with MCL primary repair and mobile-bearing change and followed up for 2 years. The patient was evaluated regarding functional capacity, pain intensity, range of motion (RoM), and quality of life. Our case showed an improvement in the functional level and the other outcomes (pain intensity and quality of life) at postoperative 2nd year when compared to the preoperative period. The wellbeing of our case in about the postop 2^nd^ year functional capacity and also other outcomes after revision surgery prompted us to continue to this surgery approach in the surgical management of similar cases that may arise thereafter.

## 1. Introduction

Unicompartmental knee replacement (UKR) is increasingly used on anteromedial knee osteoarthritis due to having a less infection rate, less invasive application, much more postoperative range of motion (RoM), and much faster healing rates than total knee replacement (TKR) [1]. Despite good postoperative results and functionality, long-term loosening rates of UKR are still much more than TKR [2]. Contrary to Kozinn and Scott [3], Pandit et al. [4] reported that the patellofemoral arthrosis, chondrocalcinosis, early age, and overweight are not anymore accepted as contraindication of using the mobile-bearing system in UKR. However, anterior cruciate ligament (ACL) and MCL insufficiency and lateral compartment arthrosis are still accepted as a contraindication for UKR application. Furthermore, the varus deformity must be fully correctable to exclude a fixed deformity. 30° varus-valgus stress X-rays and a detailed physical examination are crucial for proper indications and accurate surgical technique. Given that UKR is a prosthetic design that requires an intact MCL, the MCL insufficiency may cause an early loosening and insert dislocation.

Our UKR patient who experienced a grade 3 MCL rupture after a fall while getting on a public bus (traumatic valgus force) at postop 1st year is a valuable case to show the effects of MCL insufficiency on UKR biomechanics. Although a current case report does not describe a novel technique, according to our knowledge, there is no prior article that reports functional results of the MCL repair and the insert change after the MCL rupture in the UKR. Due to lack of a sample in this manner in the literature, we wanted to demonstrate the management and the results of quantitative functional assessments of a single UKR case with ruptured MCL.

## 2. Case Report

**Figure 1:**
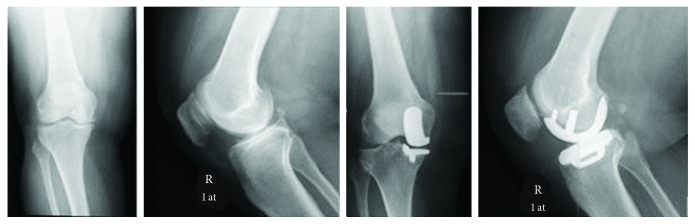
Preoperative and postoperative standing AP and lateral X-rays.

63-year-old woman who had suffered from the right medial knee pain for 5 years and was not responsive to conservative treatment was admitted to our clinics. 30° varus-valgus stress X-ray indicated that the patient had an intact MCL and LCL. After the detailed physical examination and reviewing of X-ray images, it was decided that UKR would be the most suitable option for the patient with anteromedial knee osteoarthritis. After spinal anesthesia application and sedation, the UKR surgery was performed with a standard minimal invasive midline vertical incision and a medial parapatellar approach; the patella was removed laterally but not dislocated or everted. The patient received a medial partial knee implant with a mobile-bearing insert (medium size with 4 mm thickness; Oxford®, Zimmer Biomet Inc., Warsaw, IN, USA). Following the UKR surgery (Figure 1), weight bearing was allowed as tolerated by the patient and a standard postoperative physiotherapy was started on the first postoperative day. The patient was discharged at postop 2nd day when she met the following criteria: independent ability to get dressed, to get in and out of the bed, and to sit and rise from a chair/toilet; independence in personal care; and mobilization with crutches. After discharge, a home-based exercise program was given to the patient. At postoperative follow-up, our patient acquired a full knee RoM in the postop 1st month and returned to independent daily activities without any external support in the postop 3rd month.

**Figure 2:**
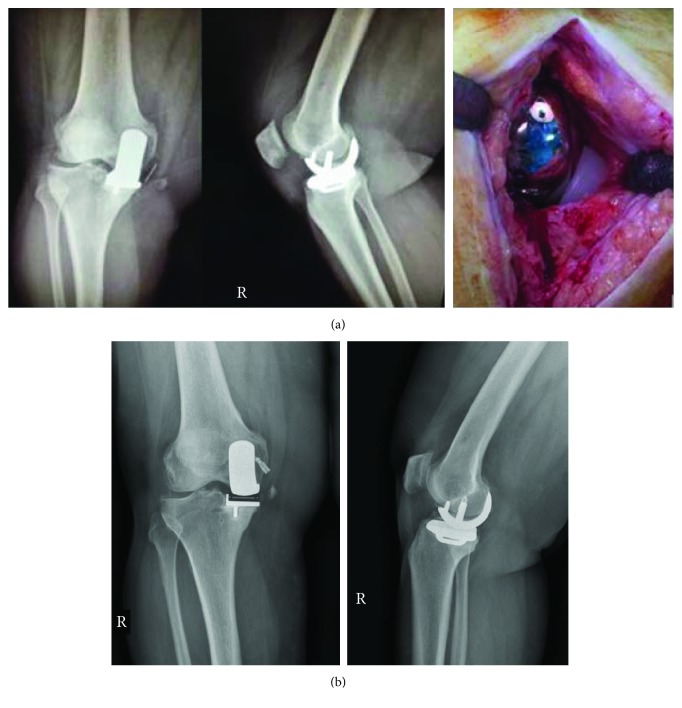
(a) X-ray and preoperative images of insert dislocation. (b) Postoperative 2nd year AP lateral standing.

At postoperative 1st year after first UKR application, the patient fell down while getting on a public bus; this caused that the right knee of the patient was exposed to the valgus force vector. After that moment, the patient heard a pop sound and felt an incredible pain that prohibited the flexion and/or extension of the medial side of the right knee. And then she was admitted to our emergency department. The first evaluation was performed, and the patient was diagnosed with a grade 3 MCL rupture and the UKR insert dislocation (Figure 2). Having completed the preoperative preparations, the patient was operated on the same day. After anesthetic administration, a surgery with a standard minimal invasive midline vertical incision and a medial parapatellar approach (to a previous incision site) was performed to change the mobile-bearing insert with the same size (medium-sized mobile-bearing insert with 4 mm thickness; Oxford®, Zimmer Biomet Inc., Warsaw, IN, USA). After having changed the mobile-bearing insert, the MCL structures were repaired and anchored to its femoral origin with a 5 mm titanium anchor. Following the surgery, weight bearing and full RoM with a hinged knee brace were allowed as tolerated by patient and a standard postoperative physiotherapy was started on the first postoperative day. Crutches were recommended for 2 to 3 weeks to enable the patient to regain a normal gait. The brace was used continuously for 4 weeks and thereafter during the day for 2 weeks. After the physiotherapy program administration, the patient was discharged at postop 1st day.

The patients were evaluated regarding pain intensity (Numeric Pain Rating Scale (NPRS)), active range of motion (RoM), and quality of life (Short-Form 12 Health Survey (SF-12 Health Survey)). Functional capacity was evaluated using the Iowa Level of Assistance Scale (ILAS), Iowa Ambulation Velocity Scale (IAVS), Hospital for Special Surgery (HSS) knee score, and Timed Up and Go (TUG) test. Rehabilitation program and outcome evaluation were conducted by one clinical physiotherapist at preoperative period (before the first UKR application), at discharge (postop 2nd day after the first UKR surgery), and at postop 2nd year (after 2 years from the MCL repair and the insert change). The evaluation results are shown in Table 1.

## 3. Discussion

Our case report is the first study that quantitatively evaluated MCL repair results in the patient with UKR. The patient showed an improvement in pain intensity, quality of life, and functional capacity scores (ILAS, TUG, and HSS) at postoperative 2nd year (2 years after the MCL repair and insert change) compared to the preoperative period.

UKR has been well documented in the literature, and the survivorship of UKR is reported to be comparable to TKR. Researchers stated that while the UKR is considered an acceptable surgical procedure, it has an underlying failure rate like any surgical procedure. The most common complication after UKR surgery is prosthetic loosening, malposition, instability, tibial collapse, and severe unexplained knee pain [2, 3, 5].

According to the study by Kim et al. [5], the insert dislocation was the most frequent complication of mobile-bearing UKR applications. They stated that the main causes of insert dislocation were as follows: the flexion-extension gap incompatibility, the insert entrapment with the femoral or tibial component, the instability due to MCL rupture, and the instability due to component loosening. Also, they reported 2 cases with MCL rupture which were treated with acute primary repair of MCL in 1576 UKR cases. Contrary to Kim et al. [5], Bergeson et al. [6] reported that aseptic loosening and persistent pain were the prominent complications of UKR as the leading cause of revision. Also, they mentioned only 2 insert dislocations and no MCL rupture among 1000 mobile-bearing UKR cases. Similar to Kim et al. [5], our UKR case with MCL rupture was treated with primary repair of MCL; however; unlike their study, our case report included the long-term functional results in addition to the treatment procedure.

Bergeson et al. [6] performed 40 revision reoperation and 16 nonrevision reoperation in 1000 UKR cases and reported an improvement in outcomes (pain, function, and clinical score of Knee Society Score (KSS)) of a total of 48 cases with UKR complication when compared to the preoperative status. Also, they pointed out that the mobile-bearing UKR with an excellent survivorship and improvement in functional outcomes of KSS is a good option for treatment of anteromedial knee osteoarthritis. Based on their 12-year experience about treatment of UKR complications, Kim et al. [5] generally suggested that conversion to TKR should be considered as an initial option in cases with UKR complications. Kim et al. [5] suggested that conversion to TKR is a favorable option and performed conversion to TKR on 58 of 89 cases with UKR complications, although they reported only 2 cases with MCL rupture in 1576 UKR cases and treated these 2 cases with acute primary repair of MCL. The results of the two previous studies are controversial about which reoperation (revision of UKR and/or conversion to TKR) should be performed after UKR complication [5–7]. Our UKR case with MCL rupture and dislocated insert was treated with revision of UKR and primary repair of MCL. Functional outcomes of our case improved at 2-year follow-up when compared to the preoperative status. In this manner, the result of our study may be useful to make a decision of appropriate surgical management in similar cases that may arise thereafter.

The MCL is the most important structure of the knee joint that contributes to the knee joint and implant stability and determines the flexion-extension gap after UKR surgery. Researchers stated that overhang of the tibial component or usage of a polyethylene insert of excessive thickness results in a weakened MCL via repetitive trauma and a chronic MCL injury [8, 9]. Also, thickness of the tibial cut depth is important to preserve the MCL insertion which closely located to the tibial plateau surface. Therefore, this should be taken into account during surgery by surgeons to secure bone-implant contact conformity and to prevent chronic injury of MCL after UKR. Gudena et al. [8] analyzed a safe overhang limit of the tibial baseplate and found out that 2 mm was the safe overhang limit and statistically significant changes have occurred when overhang was 4 mm and 6 mm. We used a medium-sized insert with 4 mm thickness for our UKR case, and there was no overhang from the tibial plateau after UKR surgery. Maes et al. [9] reported that the thicker tibial cut would result in the deep MCL defect as 9 mm cut caused 54% loss of the deep MCL insertion in their study. They suggested to use 5 mm tibial cut as an upper limit to preserve the tibial part of the deep MCL. These suggestions bolstered up our preference of tibial cut that we generally perform 5 mm tibial cut in our clinics to avoid any injury of the MCL. Given these results, the MCL rupture in our UKR case was related to the exposed valgus force vector in the knee joint other than the factors such as surgery or iatrogenic related factors.

Postoperative extremity alignment is another challenging situation that may create an environment for the MCL strain. Bearing insert dislocations are associated with proximal tibial varus greater than 5°, excessive femoral component varus or valgus, and excessive postoperative tibial slope [10]. Perkins and Gunckle [11] emphasized that the postoperative extremity axis effects on revision are needed for the UKR. They reported that the need for revision surgery increased when the postoperative tibiofemoral angle was more than 3° varus or 7° valgus. Ji et al. [10] reported that a 8 mm thick bearing insert which was changed with the 3 mm dislocated insert led to an excessive valgus at the tibiofemoral angle and so an increased lateral compartment pressure. Our case had a well-aligned extremity axis after the first UKR surgery. Also, we changed the dislocated insert with the same size; thus, the tibiofemoral alignment was not affected. Maybe this was the main factor that improved functional outcomes in our case after 2 years from insert change and MCL repair.

## 4. Conclusion

The conversion to TKR, primary MCL repair, or allograft application has been reported theoretically to cope with MCL rupture with insert dislocation in UKR cases. However, to our knowledge, there is no study about the management of insert dislocation with traumatic MCL rupture and report of functional outcomes in the UKR patient. As an uncommon situation, the traumatic MCL rupture with insert dislocation may be related to this result.

A current case report showed that the MCL repair and insert changing protocol is efficient to improve functional outcomes for 2-year follow-up. To make a general statement of our results, it needs more cases treated with our surgical procedure; however, it may be problematic due to a less probability to encounter a new case in same situation. Although we did not describe a novel technique for the treatment, the improved scores of patient 2nd year functional capacity and other outcomes after revision surgery prompted us to continue to this approach in the surgical management of similar cases that may arise thereafter. This case report may contribute to clarify the confusion in terms of choosing the best treatment option in MCL-ruptured UKR cases.

## Figures and Tables

**Table 1:** Clinical characteristic of the patient preoperatively, at discharge, and postoperative 2nd year.

Variables	Preop	Discharge	Postop 2^nd^ year
Activity NPRS (right knee)	5	2	2
Right knee RoM (degree)	118	92	110
*Functional scores*			
ILAS	18	17	24
IAVS (sec.)	10	15	10
TUG (sec.)	11	18	10
HSS (right knee)	74	61	89
*SF-12 Health Survey*			
SF-12 PCS	28.3	28.4	47
SF-12 MCS	42	55.6	60

NPRS: Numeric Pain Rating Scale; RoM: range of motion; sec.: seconds; ILAS: Iowa Level of Assistance Scale; IAVS: Iowa Ambulation Velocity Scale; HSS: Hospital for Special Surgery; TUG: Timed Up and Go; SF-12 Health Survey: Short-Form 12 Health Survey; SF-12 PCS: SF-12 Physical Health Composite Score; SF-12 MCS: SF-12 Mental Health Composite Score.

## Additional Points

*Study Location*. The current study was carried out at Dokuz Eylul University, TR-35340 Balçova, Izmir, Turkey.
